# PHN List of Reviewers

**DOI:** 10.1017/S1368980021004912

**Published:** 2022-01

The Editorial Board of *Public Health Nutrition* would like to thank the following for their contribution as peer reviewers in 2021:

Emilio Abad-Segura

Laura Aballay

Leila Abar

Vida Abedi

Joana Abou-Rizk

Taymara Abreu

Benjamin Aceves

Rachel Acton

Linda Adair

Folasade Adebayo

Theodosia Adom

Bani Aeri

Bharat B. Aggarwal

Brooke Aggarwal

George Agogo

Arif Ahmed

Junaid Ahmed

Arif Aji

Erdal Akdeniz

Blessing Akombi-Inyang

Katherine Alaimo

Ute Alexy

Cecilia Algarin

Habiba Ali

Reem Ali

Zakari Ali

Sabika Allehdan

Margaret Allman-Farinelli

Cláudia Almeida

Juliana Almeida-de-Souza

Rafael Almendra-Pegueros

Sara Almosharruf

Naser Alsharairi

Laura Alston

Uwe Altmann

Maria Alva

Seraphim Alvanides

Carlos Alvarezdardet Diaz

Mariane Alves

Teresa F. Amaral

Archana Amatya

Sophia Amenyah

Sarah Amin

Shirin Amini

Mostafa Amini Rarani

Marie-Josèphe Amiot

Ashley Amson

Annie Anderson

Giovanna Andrade

Valentina Andreeva

Tatiana Andreyeva

Dini Andrias

Mitravinda Aneesh

Stephanie Anzman-Frasca

Jessica Appleton

Katherine Appleton

Monica Arantxa Colchero

Chrisa Arcan

Mary Beth Arensberg

Gastón Ares Devincenzi

Dachi Arikpo

Nimmathota Arlappa

Soraia Arruda

Joanne Arsenault

Irma Arteaga

Richmond Aryeetey

Mireille Augustijn

Carolyn Auma

Dagfinn Aune

Parvin Ayremlou

Jose Azevedo

Fusta Azupogo

Vinicius José Baccin Martins

Rachel Bacon

Mary Bailey

Regan Bailey

Lisa Bailey-Davis

Aishat Bakre

Heather Baldwin

Khor Ban Hock

Kutha Banda

Daniel Bandoni

Larissa Baraldi

Joanna Baran

Thomas Baranowski

Tom Baranowski

Josep Xavier Barber Valles

Martina Barchitta

Salesa Barja

Pepita Barlow

Amy Barnes

Juan Ramón Barrada

Mikayla Barry

Friederike Barthels

Muhammad Khalid Bashir

Malek Batal

Katie Bates

Silvia Bauer

Imaan Bayoumi

Angela Bearth

Alyssa Beavers

Aurelie Bechoff

Eleanor Beck

Leslie M. Beitsch

Traci Bekelman

Rekia Belahsen

Bethany Bell

Winnie Bell

Alison Benbow

Vasiliki Benetou

Sara Benjamin Neelon

Christie Bennett

Tony Benson

Remco Benthem de Grave

Elling Bere

Cesar Bernal-Bravo

Wondu Garoma Berra

Ronel Beukes

Flávia Bezerra

Ilana Nogueira Bezerra

Vanessa Moraes Bezerra

Komal Bhatia

Bin-Na Bi

Piera Biccard

Erin Biehl

Nazia Binte Ali

Dowen Birkhed

Maureen M. Black

Nicole Blackstone

Miranda Blake

Sonia Blaney

Rachel Bleiweiss-Sande

Leigh Blizzard

Gail Boddy

Tara Boelsen-Robinson

Biruk Bogale

Kristy Bolton

Marialaura Bonaccio

N Bondonno

Loris Bonetti

Alison Booth

Camila Borges

Milad Borji

Emma Bouman

Christelle Bou-Mitri

Vasiliki Bountziouka

Megan Bourassa

Jean Bousquet

April Bowling

Kathryn Bradbury

Jennie Brand-Miller

Anne Lise Brantsæter

Virignia Braun

Barbara Brayner

Serge Briancon

Liliana Paula Bricarello

Eleanor Brindle

Flávia dos Santos Brito

Louise Brough

Meg Bruening

Christopher Bryant

Michael P. Burke

Luciene Burlandy

Jean Butel

Kendra Byrd

Rebecca Byrne

Anna C. Queiroz de Medeiros

Juan Francisco Cabello

Lin Cai

Nihan Çakır Biçer

Suleyman Cakiroglu

Philip Calder

Rishi Caleyachetty

Eric Calloway

Rebecca Campbell

Daniela Canella

Jonathan Cantor

Alejandra Cantoral

Giovanna Caparello

Simon Capewell

Martin Caraher

Julia Carins

Lauren Carpenter

Amy Carrad

Angela Carriedo

Alicia Carriquiry

Tanya Cassidy

Inês Castro

Itandehui Castro-Quezada

Bess Caswell

Diane Catellier

Laura Caulfield

Gustavo Cediel

Carlos Celis-Morales

Violeta Chacón

Jesse Chandler

Vicky C. Chang

Jack Chapel

Kathryn Chapman

Sarah Chaput

Karen Charlton

Helene Charreire

C. Mark Chassay

Liam Chawner

Ting Chen

Wei Chen

Joelaine Chetty

Pheak Chhoun

Tsuyoshi Chiba

Mariana Chilton

Jawad Ahmed Chishtie

Mary Foong-Fong Chong

Muhammad Abdul Baker Chowdhury

Ranadip Chowdhury

Courtney Choy

Benjamin Chrisinger

Susan Church

Christopher Cifelli

Seda Çiftçi

Marta Citelli

Jenifer Clapp

Natasha Clarke

Rafael Claro

Patricia Coffey

Juliana Cohen

Pieter Cohen

Tatiana Collese

Zoé Colombet

Kirsten Coppell

Laura Cornelsen

Ligiana Corona

Paulina Correa-Burrows

Maria Correa-Rodriguez

Caroline Costa

Silvia Costa

Simon Coulton

Cindy Crawford

David Crawford

Sandra Crispim

Nathan Critchlow

Eric Crosbie

Ruth Crowe

Sarah Crozier

Carlos Cruz Casarrubias

Katherine Cullerton

Solveig Cunningham

Cintia Curioni

Liliane da Costa

Alessandra da Silva

Danilo da Silva

Anna Dahl

Wendy Dahl

Hiroyuki Daida

Kathryn Dalrymple

Lalit Dandona

Elnaz Daneshzad

Shaonong Dang

Omar Dary

Subhasish Das

Sunanda Das

Rajat Das Gupta

Kathleen Davis

Prithwish De

Augusto Cesar De Moraes

Amanda de Moura Souza

Alessandra de Oliveira

Anniza De Villiers

Jeanne de Vries

Emily L. Deichsel

Robyn Delbridge

Hélène Delisle

R.S. Dell’Osbel

Dipti Dev

Jigna Dharod

Jennifer Di Noia

Laura di Renzo

Cristian Diaz-Velez

Melissa Dichter

Sarah Dickie

James Dillard

Lauren Dinour

Monica Dinu

Helen Dixon

Annette Dobson

Warren Dodd

Radhouene Doggui

David Doledec

Beth Dollinger

Semíramis Domene

Nan Dou

Shauna Downs

Catherine Draper

Shufa Du

Lisanne Du Plessis

Emily Duffy

Lauren Dunaway

Eleanor Dunlop

Thomas Dunn

Yashvee Dunneram

Samuel Duran

Pratibha Dwarkanath

Johanna Dwyer

Creswell Eastman

Gözde Ede

Tobias Effertz

Hassan Eini-Zinab

Maryam Ekramzadeh

Naglaa El-Abbadi

Kayi Eliacik

Charlene Elliott

Karl Emmert-Fees

Pauline Emmett

Jennifer Emond

Teri Emrich

Carla Enes

Reina Engle-Stone

Emily English

Johan Eriksson

Maijaliisa Erkkola

Getahun Ersino

Ahmad Esmaillzadeh

Camilla Estima

Stephanie Ettinger de Cuba

Charlotte Evans

Olusegun Fadare

Shiva Faghih

Susan Fairweather-Tait

Jennifer Falbe

Yu Fan

Ai-ping Fang

Meghan Farley Webb

Alisha Farris

Ana Feldenheimer da Silva

Vanessa Fernandes Davies

Rebeca Fernandez-Carrion

Erin Ferranti

Márcia Ferreira

Debbie Fetter

Giovanna M. R. Fiates

Joel Figueroa-Quiñones

Amelia B. Finaret

Sarah Jane Flaherty

Jasmine Fledderjohann

Pilar Flores

Maria de Jesus M Fonseca

Stewart Forsyth

Adrienne Forsyth

Sarah Francis

Rebecca Franckle

Deborah Frank

Gary Fraser

Maria do Carmo Freire

Gabriela Fretes

Gabriela Fretes Centurión

Rogerio Friedman

Katherine Froehlich-Grobe

Edward A. Frongillo

Jean Fry

Elizabeth Fujimori

Victor Fulgoni, III

Kamila Gabe

Piyada Gaewkhiew

Sheila Gahagan

Alisha Gaines

Pablo Gaitán-Rossi

Charis M. Galanakis

Danielle Gallegos

Marcia Galvan-Portillo

Patricia Galvez

Ryan Gamba

Kim Gans

Carmen Garcés

Aderson Garcez

Manuela García de la Hera

Teresa García-Nieto

Tany E. Garcidueñas-Fimbres

Jennifer Garner

Giovanna Gatica-Domínguez

SubbaRao Gavaravarapu

Alfredo Gea

Jerzy Gębski

Kurt Gedrich

Aulo Gelli

Jinsong Geng

Cindy George

Ekavi Georgousopoulou

Sanne Gerards

Mariette Gerber

Sarah Gerritsen

Temesgen Getaneh

Dereje G. Gete

Ceren Gezer

Delaram Ghodsi

Suparna Ghosh-Jerath

Heather Gibbs

Cheryl Gilhooly

Veronique Gingras

Tsinuel Girma

Karen Glanz

Judith R. Glynn

Justyna Godos

Rebecca Golley

Fabio Gomes

Caitlin Gomez

Fabiana Cristina Gonçalves

Cristiana Gontijo

Cristina González-Díaz

Magdalena Gornicka

Lucas Gosdin

Wendi Gosliner

Joseph L. Goulet

Fiona Graham

Sabrina Ionata Granheim

William Grant

Amanda Grech

Timothy Green

Geoff Greene

Mathilde Gressier

Jessica Grieger

Rosane Griep

Carley Grimes

Sareen Gropper

Giuseppe Grosso

Anna H. Grummon

Craig Gundersen

Himanshu Gupta

Piyush Gupta

Vidhu Gupta

Klara Gurzo

Alison Gustafson

Joanne Guthrie

Josephine Gwynn

Kyungho Ha

Rebecca Hagedorn

Amier Haidar

Sumanto Haldar

Marissa Hall

Mutinta Hambayi

Aliza Haslinda Hamirudin

Robert Hamlin

Wee Meng Han

Rhona Hanning

Karla Hanson

Polly Hardy-Johnson

Lisa Harnack

Janas Harrington

Francesca Harris

Jennifer Harris

Anna Harton

Nihal Hatipoglu

Holly Hatton-Bowers

Nicola Hawley

Christina Hecht

Valisa Hedrick

William J. Heerman

Rebecca Heidkamp

Mirjam Heinen

Azita Hekmatdoost

Rebecca Helmreich

Helen Hermsdorff

Juan José Hernández Morante

Akram Hernández-Vásquez

Kirsten Herrick

Isabelle Herter-Aeberli

Julie Hess

Tim Hess

Frances Hillier-Brown

Melanie Sberna Hinojosa

Kajal Hirani

Kalle Hirvonen

Jody Hoenink

Richard Hoffman

Ingrid Hoffmann

Bruce Hollis

Graham Horgan

Jean-Luc Hornick

Paula Horta

Susan Horton

Moyazzem Hossain

Hedayat Hosseini

Firoozeh Hosseini-Esfahani

M. Katherine Hoy

Amber Hromi-Fiedler

Dongsheng Hu

Sophia Hua

Jianping Huang

Jin Huang

Liping (Polly) Huang

Yi-Chen Huang

Jorg Huber

Maria Celia Hughes

Roger Hughes

Renee Hughner

Monica Hunsberger

Sandra Hunziker

Martin Hushie

Laila Hussein

Sungwoo Hwang

Natalie K. Hyde

Scott Ickes

Alessandro Iellamo

Carolyn Ievers-Landis

Kristine Illøkken

Ana Irache

Andrew Irish

Midori Ishikawa

Lígia Isoni Auad

Leila Itani

Toshiyuki Iwahori

Masanori Iwasaki

Betty Izumi

Madalina Jacota

Sara Jaeger

Patricia Jaime

Zeina Jamaluddine

Yasaman Jamshidinaeini

Kathryn Janda

Megan Jarman

Alfred Jatho

Maria Elena Jefferds

Jørgen Jensen

Melissa Jensen

Joshua Jeong

Theresa Jeremias

Jo Jewell

Peng Jia

Kari Johansson

Hope Johnson

Kimberly Johnson

William Johnson

Glenys Jones

Jessica Jones-Smith

Irmgard Jordan

Chantal Julia

Shinyoung Jun

Ju Young Jung

Filippa Juul

Russell Kabir

Juliana Kain

Shubhada Kanani

Gagandeep Kang

Adam Kantanista

Deksha Kapoor

Ridhima Kapoor

Nena Karavasiloglou

Lutfiye Karcioglu Batur

Şaban Kargiglioglu

Saujanya Karki

Tilakavati Karupaiah

Christina Kasprzak

Suna Kassier

Yoshiko Kato

Kendra Kattelmann

Manmeet Kaur

Justine A Kavle

Yuhei Kawano

Sanin Kazi

Roya Kelishadi

Bridget Kelly

Deborah Kennedy

Erica Kenney

George Kent

Marko Kerac

Mathilde Kersting

Alev Keser

Ahmed Gharib Khamis

Jahidur Rahman Khan

Nizamuddin Khan

Neha Khandpur

Muhammad Nasir Khan Khattak

Ban Khor

Pramod Khosla

Hyesook Kim

Jihye Kim

Kijoon Kim

Kirang Kim

Mi Kyung Kim

Judith Kimiwe

Christian King

Sara Kirk

Knut-Inge Klepp

Susanne Kobel

Stephen Kodish

Marjukka Kolehmainen

Maria Koleilat

Joel Komakech

Kai Ling Kong

Amanda Kopetsky

Tim Korevaar

Lukas Kornher

Anastassia Kossioni

Albert Koulman

Dimitris Koutoukidis

Matina Kouvari

Tatsuya Koyama

Slawomir Koziel

Nancy F. Krebs

Susan Krebs-Smith

Carolin Krems

Greta Krešić

Eleonore Kretz

Anja Kroke

Ozge Kucukerdonmez

Praveen Kumar

Tony Kuo

Emil Kupek

Debbie Kupolati

Konsita Kuswara

Michael R. La Frano

Ronald Labonte

Marie-Ève Labonté

Elisa Maria Lacerda

Fernando Lamarca

Abera Lambebo

Alicia Landry

Matthew Landry

Melissa Lane

Matthew A. Lapierre

Nicole Larson

Fiona Lavelle

Ziva Lavrisa

Ha Le

Phung Le

Tashara Leak

Miriam P. Leary

Madison N. LeCroy

Charlotte Lee

Myoungsook Lee

Misgan Legesse

Reetta Lehto

Maria Leite

Herman Lelieveldt

Lucia Leone

Laura Lessard

Seven Levy

Joshua R. Lewis

Meron Lewis

Anne-Marie Lewis-Mikhail

Yan Li

Caitlin Liddelow

Djin Gie Liem

Nanna Lien

Angela Liese

Juliana Lignani

Qian Lin

Tiffany Lin

Rebecca Lindberg

Anna Karin Lindroos

Anna Lipert

Cinthia Lisboa

Natalie Lister

Yi-Hsuan Liu

Zhao-Min Liu

Geoffrey Livesey

Katherine Livingstone

Margarida Liz Martins

Kenneth Lo

Lindsey Locks

Antje Löffler

Mariana Lopes

Nancy Lopez-Olmedo

Alissia Lourme-Ruiz

Penny Love

Hui Luan

Patricia Lucas

Mary-Jon Ludy

Juan Luis Gonzalez-Pascual

Nyati Lukhanyo

LH Lumey

Gabriella Luongo

Shengmin Lv

Guansheng Ma

Xiaonan Ma

Márcia Machado

Una MacIntyre

Sally Mackay

Kristine Madsen

Damian Maganja

Zhila Maghbooli

Roberta Magnano San Lio

Emmanuella Magriplis

Catherine Mah

Matthieu Maillot

Laís Mais

Hazreen Majid

May Mak

Kevin Maki

Akbar Malekshah

Kenneth Maleta

Shelly Malik

Vasanti S. Malik

Marcio Correa Mancini

Swetha Manohar

Liina Mansukoski

Limei Mao

Dirce Marchioni

Jessica Marcinkevage

Barrie Margetts

Aline Mariath

Bruna Marmett

Iva Marques-Lopes

Grace Marquis

Fernanda Marrocos Leite

Maria Dolores Marrodán

Stephanie Martin

Nerea Martin-Calvo

Edson Zangiacomi Martinez

Euridice Martinez Steele

Alba Martínez-García

Francisco Martin-Lujan

Yves Martin-Prevel

Carla Martins

Aline Martins de Carvalho

Gabriel Masset

Travis Masterson

Marco Mastroeni

Gina Marie Mathew

Mariana Mathias

Maria Gabriela Matias de Pinho

Mai Matsumoto

Josiemer Mattei

Katarina Matthes

Anna Mattioli

Louisa Matwiejczyk

Kellie Mayfield

Mónica Mazariegos

Mohsen Mazidi

Artur Mazur

Xikombiso Gertrude Mbhenyane

Teresia Mbogori

Tulisiwe Mbombo-Dweba

Mduduzi Mbuya

Janelle McAlpine

Kate McBride

Mary McCann

Amanda McClain

Brittany McCormick

Christine McDonald

Thérèse McDonnell

Ryan McGrath

Lynn McIntyre

Nicola McKeown

Christina McKerchar

Rachael McLean

Taylor McLinden

Gabriella M. McLoughlin

Jade McNamara

Kondolot Meda

Fernanda Mediano

Jose Medina-Inojosa

Kirsten Mehlig

Sarah Meiklejohn

Tefera Mekonnen

Juana Meléndez Torres

Fei Men

Larissa Mendes

Kathryn Merckel

Mirella Meyer-Ficca

Agnieszka Micek

Moscho Michalopoulou

Cedric Middel

José Mill

Daniel Miller

Brandy-Joe Milliron

Catherine Milte

Jezid Miranda

Julia Mirotta

Khadijeh Mirzaei

Masoud Mirzaei

Gita Mishra

Perpetua Modjadji

Fatemeh Mohammadi-Nasrabadi

Devi Mohan

Zalilah Mohd Shariff

Morgana Mongraw-Chaffin

Carlos Monteiro

Rob Moodie

Sally Moore

Sajjad Moradi

Milena Moraes Ferreira

Maria Morales Suárez-Varela

Thereza Moreira

Emily Morgan

Belinda Morley

Katrina Moss

Janaína Motta

Jean-Claude Moubarac

Sarah Mounsey

Claire Mouquet-Rivier

Fabiana Moura

Stamatis Mourtakos

Dariush Mozaffarian

Megan Mueller

Gudani Mukoma

Manfred James Müller

Angela Mulligan

Sergio Muñoz

Mary Murimi

Blain Murphy

Sohail Mushtaq

Siti Muslimatun

Maurice Mutisya

Antonina Mutoro

Candice Myers

Emily A. Myers

Muzi Na

Seyed M. Nachvak

Simeon Nanama

Jacob Napieralski

Anuradha Narayan

Marcus V. S. Nascimento

Nenad Naumovski

Eva Maria Navarrete-Muñoz

Pantea Nazeri

Elizabeth Neale

Michael Nelson

Jerusha Nelson-Peterman

Marion Nestle

Merryn Netting

Paulo Augusto Neves

SeeHoe Ng

Yandisa Ngqangashe

Kim Nguyen

Phuong Nguyen

Khristopher Nicholas

Sophie Nicklaus

Mary Nicolaou

Sara Nieuwoudt

Eduardo Nilson

Li Niu

Lawrence Achilles Nnyanzi

Laurence J. Nolan

Stacia Nordin

Jennifer Norman

Paula Normando

Tom Norris

Tanita Northcott

Kate Northstone

Paulina Nowicka

Anne Nugent

Ali Nurshad

Chijioke Nwosu

Brietta Oaks

Omar Obeid

Angelica Ochoa-Avilés

Sophie Ochola

Lauren O'Connor

Aisling O'Donnell

Ropo Ebenezer Ogunsakin

Julius Okello

Nagako Okuda

Beatrice Olack

Barbara Olendzki

Jose Oliveira

Klebya Oliveira

Mette Olsen

Nanna Olsen

Naiá Ortelan

Aaron J. O'Sullivan

Joost Oude Groeniger

Ousmane Ouedraogo

Yi-fei Ouyang

Burcu Ates Ozcan

Ifeoma Ozodiegwu

Patricia Padilha

Cristina Palacios

Poliana Palmeira

Demosthenes Panagiotakos

Yanni Papanikolaou

Vitor Paravidino

Niyati Parekh

Sohyun Park

Haley Parker

Kate Parker

Keryn Pasch

Yahya Pasdar

Valéria Passos

Rosario Pastor

Silvia Pastorino

Anisha Patel

Nehali Patel

Emma Patterson

Joanne Patterson

Elise Pauzé

Lilia Pedraza-Zamora

Evan D Peet

Dolores Penafiel

W. Todd Penberthy

Nicholas Pennings

Mary Penny

Marcos Pereira

Kingsley Pereko

Rafael Pérez-Escamilla

Carolina Perez-Ferrer

Carmen Perez-Rodrigo

Ana Seselja Perisin

Milene Pessoa

Joshua Petimar

Janina Petkeviciene

John Pettifor

Simone Pettigrew

Uyen T. X. Phan

Kirthee Pillay

Anna Christina Pinheiro

Elisabete Pinto

Tito Pizarro

Ana Poblacion

Peggy Policastro

Christina Pollard

Jane Polsky

Sonia Pombo-Rodrigues

Jennifer Pomeranz

Michael Pomeroy

Janet Poppendieck

Tanja Poulain

Makan Pourmasoumi

Elaine Power

Thomas Power

Rebecca Pradeilles

Igor Pravst

Matt A. Price

Flavia Prodam

Flavia Prodram

Jennifer Protudjer

Rachel Prowse

Hildegard Przyrembel

Viliami Puloka

Digna Purwaningrum

Nell Putnam-Farr

Diane Putnick

Matin Qaim

Phaik Ling Quah

Daiana Quintiliano

Fatemeh Khan Rabiee

Amanda Raffoul

Hasinur Rahaman Khan

Mehdi Rahimian

Kim Raine

Abdolhalim Rajabi

Anna K. Ramnemark

Patricia Ramos

Vasco Ramos

Juel Rana

Natalie Rangelov

Nalini Ranjit

Shobha Rao

Ilana Raskind

Neha Rathi

Svetlana Ratushnyak

Divya Ravindranath

Carola Ray

Qaisar Raza

Helena Real

Sophie Reale

Elisabetta Recine

Layton Reesor

Michael Reichenheim

Marla Reicks

Gláubia Relvas

Yanjun Ren

Brandon J. Restrepo

Anabelle Retondario

Marcela Reyes

Cecília Cláudia Ribeiro

Camila Ricardo

Fulvio Ricceri

Megan Richards

Katie Ricketts

Bradley Ridoutt

Natalie Riediger

Eric Rimm

Sofia Rincón Gallardo Patiño

Jesús Rivera Navarro

Ella Robinson

Brenda Robles

Paula Robson

Shannon Robson

Paola Rodrigo-Gallardo

Paulo Rodrigues

Alexander Rodriguez

Celia Rodriguez-Perez

Stephanie Rogus

Sascha Rohn

Federico Roncarolo

Anna J. Rose

Donald Rose

Luis Rosero Bixby

Alice Rosi

Tanti Irawati Rosli

Jessica Rothstein

Emily Rousham

Maisie Rowland

Rajshri Roy

Diana Rubin

Dorte Ruge

Miguel Ruiz-Canela

Emily Ruppert

Cherie Russell

Joanna Russell

Rienna Russo

Femke Rutters

Kate Sadler

Richard Sadler

Sanjoy Saha

Amanda Sainsbury

Emma Sainsbury

Asma Salari-Moghaddam

Amin Salehi-Abargouei

Rosana Salles-Costa

Maria Reyna Sámano

Jennifer Sanchez-Flack

Rajan Sankar

Débora Santos

Francisco Sarabia-Sanchez

Bahareh Sarmadi

Isabela Sattamini

Cláudia Saunders

Mateja Savoie-Roskos

Amy Saxe-Custack

Ellen Schafer

Miriam Schiff

Virginia Schmied

Whitney Schott

Stine Schramm

Natalie Scime

Stephanie Scott

Maree Scully

Susy Sebayang

Gabriela Sebokova

Ana Segall-Côrrea

Gina Segovia-Siapco

Laury Sellem

Marjanne Senekal

Hafiz Shahbaz

Pamela Shapiro

Mohit Sharma

Jesse Sheftel

Minxue Shen

Wan Shen

Natalie Shenker

Justin C Sherwin

Weijia Shi

Zumin Shi

Sangeetha Shyam

Soraya Siabani

Rosely Sichieri

Renuka Silva

Maria-Raquel Silva

Maria Asuncion Silvestre

Anja Simmet

Ana Paula Simões-Wüst

Prashant Kumar Singh

Shambhavi Singh

Chelsea Singleton

Anju Pradhan Sinha

Sheela Sinharoy

Shashikala Sivapathy

Sheila Skeaff

Joyce Slater

Jessica Smith

Matthew R. Smith

Kailey Snyder

Cynthia Sob

Karen Sokal-Gutierrez

Jessica Soldavini

Noel Solomons

Shawn Somerset

Christine Sommer

Yi Song

YoonJu Song

Diana Soria-Contreras

Kimmo Sorjonen

Joshua Sparks

Thalia Sparling

Katherine Speirs

Suzanne Spence

Rebecca Spencer

Marine Spiteri

Chittur Srinivasan

Fiona Stacey

Rosemary Stanton

Norbert Stefan

Steen Stender

Lena Stephens

Samara Sterling

Nelia Steyn

Barbara Stoecker

Genevieve Stone

Nanette Ströbele-Benschop

Josine Stuber

Christopher Sudfeld

Karine Suissa

Claire Sulmont-Rossé

Guangwen Sun

Jiang-Wei Sun

Yongye Sun

Sumathi Swaminathan

Rina Swart

Elize Symington

Mehroosh Tak

Hidemi Takimoto

Alison Talbert

Sameera Talegawkar

Gourab Raj Talukdar

Kok Hian Tan

Sze-Yen Tan

Chin Xuan Tan

Hilary Tanenbaum

Alice Tang

Kathy Tannous

Sherry Tanumihardjo

Menghua Tao

Valerie Tarasuk

Anna Taraszewska

Carla Tarazona

Evangelia Tavoulari

Ines Taylor

Rachael Taylor

Steve Taylor

Ernestine Tecklenburg

Weiping Teng

Céline Termote

Paola Tessari

Alexander Testa

Ramona Teuber

Amanda Thompson

Katie Thompson

Jessica Thomson

Anne Thorndike

Andrew Thorne-Lyman

Lukar Thornton

Karen Thorpe

Maree Thorpe

Saurabh Thosar

Anne Marie Thow

Arnold Timmer

Lesley Tinker

Lire Tirore

Christiana Rialine Titaley

Sergio Tobon Tobon

Ulla Toft

Estefania Toledo

Yasutake Tomata

Cecilia Tomori

Tammy Tong

Susan Torres

Alison Tovar

Maret Traber

Pierre Traissac

Thach Tran

Ursula Trübswasser

Emily Truman

Paula Trumbo

Claire N. Tugault-Lafleur

Lindsey Turner

Aida Turrini

Douglas Twenefour

Wayne Twine

Bryan Tysinger

Jalal Uddin

Mishel Unar-Munguía

Mudita Upadhyaya

Jacqueline Urban

Muhammed A. Usman

Chioma Uzoigwe

Flavio Valente

Louise van den Berg

Paige van der Pligt

Jolieke van der Pols

Averalda Van Graan

Linde van Lee

Lana Vanderlee

Jeremy Vanhelst

Maria Ines Varela-Silva

Hernando Vargas-Uricoechea

Jithin Varghes

Amy Vassallo

Emilia Vassilopoulou

Juliana Vaz

Gabriela M. Vedovato

Sonia Venancio

Angeliek Verdonschot

Eliseu Verly-Jr

Letícia Vertulli

Lydi-Anne Vézina-Im

Rodrigo Vianna

Helen Vidgen

Reinhold Vieth

Florent Vieux

Mireya Vilar-Compte

Salvador Villalpando

Anthony Villani

Denis Vinnikov

Francesco Visioli

Marina Visser

Rachel Vollmer

Sebastian Vollmer

Peter von Philipsborn

Syed Naimul Wadood

Yukiko Wagatsuma

Ruth Wallace

Corinna Walsh

Christine Walters

Dongqing Wang

Vivian Wang

Sarah Warkentin

Petra Warschburger

Lydiah Waswa

Cody Watling

Rosanna Watowicz

Wendy Watson

Christina Watts

Bernhard Watzl

Karen Webb

Jonathan Wells

Jean Welsh

Marianna Wetherill

Lauren White

Vanessa White-Barrow

Lisa Whitehead

Susan Whiting

Clare Whitton

Michael Widener

Simon Wieser

Cervantée Wild

Parke Wilde

Peter Willatts

Anne Williams

Susan Williams

Hendig Winarno

Pattanee Winichagoon

Megan Winkler

James Wirth

Anna Maria Witkowska

Chantell Witten

Julia Wolfson

Martin Wong

Jennifer Woo-Baidal

Julie Woods

Charlotte Wright

Feitong Wu

Jiang-Nan Wu

Qijun Wu

Qiong Wu

Wei-Te Wu

Rebecca Wyse

Fei Xu

Tao Xu

Hong Xue

Nurcan Yabanci Ayhan

Najat Yahia

Siddika Songul Yalcin

Sermin Yalin Sapmaz

Miwa Yamaguchi

Alice Yan

Weili Yan

Valentine Yanchou Njike

Seungmi Yang

Wai Yew Yang

Wenfang Yang

Mary Yannakoulia

Amy Yau

Haci Omer Yilmaz

Sunmi Yoo

Leanne Young

Sera Young

Staci Young

Zhiping Yu

Apolinaras Zaborskis

Meysam Zarezadeh

Laura Zatz

Augustin Nawidimbasba Zeba

Rena Zelig

Fang-fang Zeng

Dongfeng Zhang

Jianduan Zhang

Qi Zhang

Yan Zhang

Yanbo Zhang

Lixia Zhao

Yuqing Zheng

Long Zhou

Nida Ziauddeen

Suzanna Zick

Meghan Zimmer

A. Zink

Christina Zorbas

Luisa Zuccolo

